# Bis(μ-*N*,*N*′-di-3-pyridyl-2,6-pyridine-2,6-dicarboxamide-κ^2^ *N*:*N*′)bis[dibrom­ido­mercury(II)] *N*,*N*-dimethyl­formamide disolvate

**DOI:** 10.1107/S1600536808028754

**Published:** 2008-09-13

**Authors:** Li-hua Huang, Jie Wu

**Affiliations:** a Department of Chemistry, Zhengzhou University, Zhengzhou 450052, People’s Republic of China

## Abstract

In the dinuclear centrosymmetric title complex, [Hg_2_Br_4_(C_17_H_13_N_5_O_2_)_2_]·2C_3_H_7_NO, the Hg^II^ atom is coordinated by two Br atoms and two N atoms from two different ligands in a distorted tetra­hedral geometry. The solvent mol­ecule is linked to the 28-atom ring by two hydrogen bonds.

## Related literature

For related literature, see: Baer *et al.* (2002 ▶); Chae *et al.* (2004 ▶ and references cited therein); Qin *et al.* (2003 ▶).
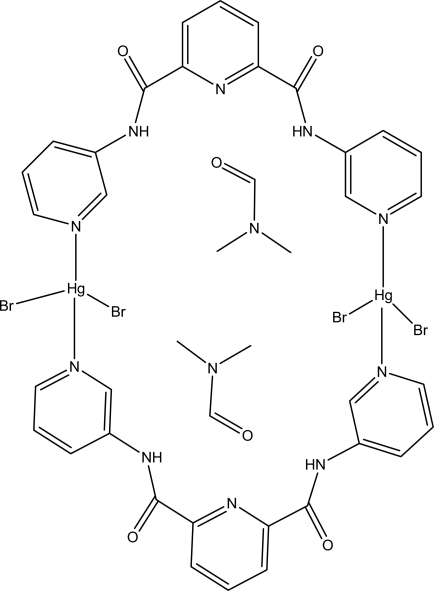


## Experimental

### 

#### Crystal data

[Hg_2_Br_4_(C_17_H_13_N_5_O_2_)_2_]·2C_3_H_7_NO
*M* *_r_* = 1505.62
Triclinic, 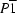

*a* = 7.7609 (16) Å
*b* = 12.267 (3) Å
*c* = 13.296 (3) Å
α = 92.27 (3)°
β = 105.82 (3)°
γ = 104.07 (3)°
*V* = 1173.7 (4) Å^3^
*Z* = 1
Mo *K*α radiation
μ = 10.00 mm^−1^
*T* = 293 (2) K
0.20 × 0.18 × 0.17 mm

#### Data collection

Rigaku Saturn724 diffractometer
Absorption correction: multi-scan (*CrystalClear*; Rigaku/MSC, 2006 ▶) *T*_min_ = 0.240, *T*_max_ = 0.281 (expected range = 0.156–0.183)
14249 measured reflections
5337 independent reflections
4364 reflections with *I* > 2σ(*I*)
*R*_int_ = 0.032

#### Refinement

*R*[*F* ^2^ > 2σ(*F* ^2^)] = 0.037
*wR*(*F* ^2^) = 0.067
*S* = 1.03
5337 reflections
299 parameters
2 restraints
H atoms treated by a mixture of independent and constrained refinement
Δρ_max_ = 0.61 e Å^−3^
Δρ_min_ = −0.71 e Å^−3^

### 

Data collection: *CrystalClear* (Rigaku/MSC, 2006 ▶); cell refinement: *CrystalClear*; data reduction: *CrystalClear*; program(s) used to solve structure: *SHELXS97* (Sheldrick, 2008 ▶); program(s) used to refine structure: *SHELXL97* (Sheldrick, 2008 ▶); molecular graphics: *SHELXTL* (Sheldrick, 2008 ▶); software used to prepare material for publication: *SHELXTL*.

## Supplementary Material

Crystal structure: contains datablocks global, I. DOI: 10.1107/S1600536808028754/ng2488sup1.cif

Structure factors: contains datablocks I. DOI: 10.1107/S1600536808028754/ng2488Isup2.hkl

Additional supplementary materials:  crystallographic information; 3D view; checkCIF report

**Fig. 3.**
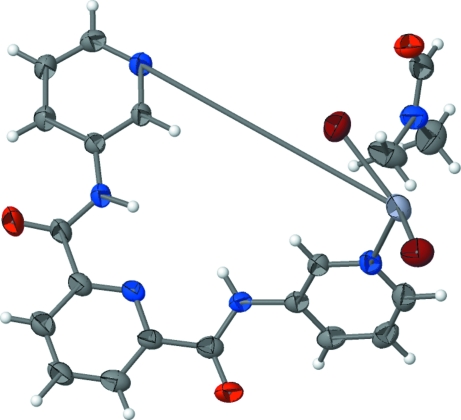
Supplementary figure.

Enhanced figure: interactive version of Fig. 3

## Figures and Tables

**Table 1:** Hydrogen-bond geometry (Å, °)

*D*—H⋯*A*	*D*—H	H⋯*A*	*D*⋯*A*	*D*—H⋯*A*
N4—H22⋯O3^i^	0.859 (10)	2.08 (2)	2.891 (5)	157 (4)
N2—H21⋯O3^i^	0.856 (10)	2.34 (2)	3.076 (5)	144 (3)
N2—H21⋯N3	0.856 (10)	2.25 (4)	2.685 (5)	111 (3)

Symmetry code: (i) 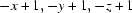.

**Fig. 1.**
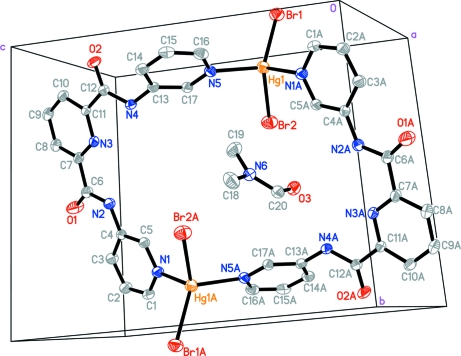
View of the title complex, showing the labeling of the non-H atoms and 30% probability ellipsolids. H atoms have been omitted.

**Fig. 2.**
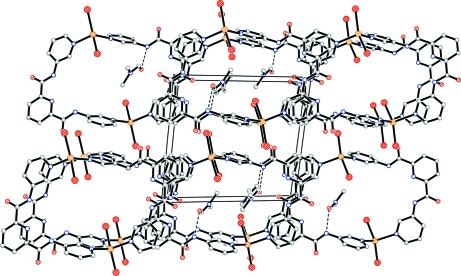
A view of the crystal packing along the a axis. Hydrogen bonds are shown as dashed lines.

## supplementary crystallographic information

### Comment

Metal-organic frameworks (MOFs) with microporous is currently of great interest because of their interesting structures and potential applications. So far, some interesting microporous MOFs have been documented (Chae *et al.* 2004, and references cited therein). One of the popular strategies to fabricate such compounds is to design the rigid ligands which have the ability to bridge the metal centers with big ring by utilizing their coordination sites. The rigid conjugated clamp-like multi-pyridine ligand *N*,*N*'-bis(pyridin-3-yl)-2,6-pyridinedicarboxamide has been known as a good candidate in the construction of MOFs with big ring (Qin *et al.* 2003; Baer *et al.* 2002). In this work, we selected this ligand as linker, generating a new coordination complex, [HgIIBr2(C17N5O2)](DMF), (I), which is reported here. In compound (I) each HgII atom is four-coordinated by two N atoms from two ligands and two Br atoms in a distorted tetrahedral coordination sphere (Fig. 1). The two HgII atoms are bridged with two *N*,*N*'-bis(pyridin-3-yl)-2,6-pyridinedicarboxamide ligands to form a microporous MOFs with 28-number ring. The neighbouring units are linked by the interactions to form a two-dimensional network (Fig. 2) and hydrogen bonds arising between the DMF and *N*,*N*'-bis(pyridin-3-yl)-2,6-pyridinedicarboxamide ligand (Table 2) complete the structure.

### Experimental

The ligand *N*,*N*'-bis(pyridin-3-yl)-2,6-pyridinedicarboxamide (0.05 mmol, 0.016 g) in DMF (5 ml) was added dropwise to a solution of HgBr2 (0.1 mmol, 0.036 g) in methanol (3 ml). The precipitate was filtered and the resulting solution was allowed to stand at room temperature in the dark. After one week good quality colorless crystals were obtained and dried in air.

### Crystal data

**Table:**

[Hg_2_Br_4_(C_17_H_13_N_5_O_2_)_2_]·2C_3_H_7_NO	*Z* = 1
*M**_r_* = 1505.62	*F*(000) = 712
Triclinic, *P*1	*D*_x_ = 2.130 Mg m^−^^3^
*a* = 7.7609 (16) Å	Mo *K*α radiation, λ = 0.71073 Å
*b* = 12.267 (3) Å	Cell parameters from 3306 reflections
*c* = 13.296 (3) Å	θ = 3.2–27.5°
α = 92.27 (3)°	µ = 10.00 mm^−^^1^
β = 105.82 (3)°	*T* = 293 K
γ = 104.07 (3)°	Prism, colourless
*V* = 1173.7 (4) Å^3^	0.20 × 0.18 × 0.17 mm

### Data collection

**Table:**

Rigaku Saturn724 diffractometer	5337 independent reflections
Radiation source: fine-focus sealed tube	4364 reflections with *I* > 2σ(*I*)
graphite	*R*_int_ = 0.032
Detector resolution: 28.5714 pixels mm^-1^	θ_max_ = 27.5°, θ_min_ = 3.2°
dtprofit.ref scans	*h* = −10→10
Absorption correction: multi-scan (CrystalClear; Rigaku/MSC, 2006)	*k* = −15→15
*T*_min_ = 0.240, *T*_max_ = 0.281	*l* = −17→17
14249 measured reflections	

### Refinement

**Table:**

Refinement on *F*^2^	Primary atom site location: structure-invariant direct methods
Least-squares matrix: full	Secondary atom site location: difference Fourier map
*R*[*F*^2^ > 2σ(*F*^2^)] = 0.037	Hydrogen site location: inferred from neighbouring sites
*wR*(*F*^2^) = 0.067	H atoms treated by a mixture of independent and constrained refinement
*S* = 1.03	*w* = 1/[σ^2^(*F*_o_^2^) + (0.0274*P*)^2^] where *P* = (*F*_o_^2^ + 2*F*_c_^2^)/3
5337 reflections	(Δ/σ)_max_ < 0.001
299 parameters	Δρ_max_ = 0.61 e Å^−^^3^
2 restraints	Δρ_min_ = −0.71 e Å^−^^3^

### Special details

**Table:**

Geometry. All e.s.d.'s (except the e.s.d. in the dihedral angle between two l.s. planes) are estimated using the full covariance matrix. The cell e.s.d.'s are taken into account individually in the estimation of e.s.d.'s in distances, angles and torsion angles; correlations between e.s.d.'s in cell parameters are only used when they are defined by crystal symmetry. An approximate (isotropic) treatment of cell e.s.d.'s is used for estimating e.s.d.'s involving l.s. planes.
Refinement. Refinement of *F*^2^ against ALL reflections. The weighted *R*-factor *wR* and goodness of fit *S* are based on *F*^2^, conventional *R*-factors *R* are based on *F*, with *F* set to zero for negative *F*^2^. The threshold expression of *F*^2^ > σ(*F*^2^) is used only for calculating *R*-factors(gt) *etc*. and is not relevant to the choice of reflections for refinement. *R*-factors based on *F*^2^ are statistically about twice as large as those based on *F*, and *R*- factors based on ALL data will be even larger.

### Fractional atomic coordinates and isotropic or equivalent isotropic displacement parameters (Å^2^)

**Table:**

	*x*	*y*	*z*	*U*_iso_*/*U*_eq_	
Hg1	0.35413 (3)	0.143038 (15)	0.325732 (13)	0.04901 (8)	
Br1	0.37301 (7)	−0.05158 (4)	0.27313 (4)	0.05602 (14)	
Br2	0.63842 (7)	0.30874 (4)	0.39232 (4)	0.06222 (15)	
O1	0.8838 (6)	0.5153 (3)	1.1145 (3)	0.0790 (12)	
O2	0.1707 (5)	0.0697 (3)	0.7966 (2)	0.0565 (9)	
N1	0.8086 (5)	0.7906 (3)	0.8251 (3)	0.0394 (8)	
N2	0.7259 (5)	0.5300 (3)	0.9465 (3)	0.0399 (9)	
N3	0.5289 (5)	0.3133 (3)	0.9248 (2)	0.0342 (8)	
N4	0.3039 (5)	0.2237 (3)	0.7291 (3)	0.0375 (8)	
N5	0.1886 (5)	0.1582 (3)	0.4441 (3)	0.0388 (8)	
C1	0.9457 (6)	0.8600 (4)	0.9012 (3)	0.0410 (11)	
H1	0.9948	0.9335	0.8888	0.049*	
C2	1.0151 (6)	0.8257 (4)	0.9966 (3)	0.0458 (11)	
H2	1.1084	0.8766	1.0485	0.055*	
C3	0.9496 (6)	0.7169 (4)	1.0172 (3)	0.0429 (11)	
H3	0.9979	0.6935	1.0822	0.051*	
C4	0.8082 (6)	0.6423 (3)	0.9377 (3)	0.0323 (9)	
C5	0.7417 (6)	0.6847 (3)	0.8436 (3)	0.0356 (10)	
H5	0.6454	0.6369	0.7907	0.043*	
C6	0.7650 (6)	0.4739 (4)	1.0323 (3)	0.0423 (11)	
C7	0.6516 (6)	0.3533 (3)	1.0186 (3)	0.0376 (10)	
C8	0.6773 (7)	0.2900 (4)	1.1030 (3)	0.0466 (12)	
H8	0.7648	0.3211	1.1670	0.056*	
C9	0.5708 (7)	0.1802 (4)	1.0900 (3)	0.0477 (12)	
H9	0.5851	0.1356	1.1451	0.057*	
C10	0.4421 (6)	0.1373 (4)	0.9935 (4)	0.0421 (11)	
H10	0.3679	0.0633	0.9827	0.050*	
C11	0.4256 (6)	0.2061 (3)	0.9135 (3)	0.0353 (10)	
C12	0.2874 (6)	0.1602 (3)	0.8079 (3)	0.0380 (10)	
C13	0.1959 (6)	0.1874 (3)	0.6234 (3)	0.0352 (10)	
C14	0.0045 (6)	0.1485 (3)	0.5954 (4)	0.0418 (11)	
H14	−0.0582	0.1444	0.6462	0.050*	
C15	−0.0910 (6)	0.1160 (4)	0.4913 (4)	0.0468 (12)	
H15	−0.2198	0.0901	0.4707	0.056*	
C16	0.0035 (7)	0.1218 (4)	0.4176 (4)	0.0463 (11)	
H16	−0.0629	0.0998	0.3472	0.056*	
C17	0.2825 (6)	0.1910 (3)	0.5445 (3)	0.0343 (9)	
H17	0.4111	0.2175	0.5627	0.041*	
H22	0.396 (4)	0.283 (2)	0.745 (3)	0.044 (13)*	
H21	0.642 (4)	0.491 (3)	0.8924 (19)	0.039 (12)*	
O3	0.4162 (5)	0.5774 (3)	0.2838 (2)	0.0568 (9)	
N6	0.1662 (6)	0.5374 (3)	0.3463 (3)	0.0465 (10)	
C18	−0.0003 (7)	0.5662 (5)	0.3550 (4)	0.0653 (15)	
H18A	−0.0186	0.6282	0.3152	0.098*	
H18B	0.0132	0.5875	0.4275	0.098*	
H18C	−0.1054	0.5019	0.3280	0.098*	
C19	0.2125 (9)	0.4419 (4)	0.3969 (4)	0.0719 (17)	
H19A	0.3199	0.4287	0.3813	0.108*	
H19B	0.1099	0.3759	0.3715	0.108*	
H19C	0.2385	0.4576	0.4716	0.108*	
C20	0.2735 (8)	0.5963 (4)	0.2962 (3)	0.0503 (12)	
H20A	0.2392	0.6583	0.2671	0.060*	

### Atomic displacement parameters (Å^2^)

**Table:**

	*U*^11^	*U*^22^	*U*^33^	*U*^12^	*U*^13^	*U*^23^
Hg1	0.06762 (15)	0.04080 (12)	0.03641 (11)	0.01450 (9)	0.01118 (9)	0.00594 (8)
Br1	0.0631 (3)	0.0433 (3)	0.0679 (3)	0.0189 (2)	0.0251 (3)	0.0047 (2)
Br2	0.0546 (3)	0.0547 (3)	0.0623 (3)	0.0063 (3)	0.0000 (3)	0.0042 (3)
O1	0.105 (3)	0.053 (2)	0.039 (2)	−0.011 (2)	−0.016 (2)	0.0158 (17)
O2	0.067 (2)	0.0389 (18)	0.049 (2)	−0.0133 (17)	0.0173 (17)	0.0083 (16)
N1	0.044 (2)	0.035 (2)	0.0344 (19)	0.0063 (17)	0.0071 (17)	0.0039 (16)
N2	0.047 (2)	0.037 (2)	0.0269 (19)	0.0029 (18)	0.0026 (17)	0.0033 (17)
N3	0.041 (2)	0.0331 (19)	0.0315 (19)	0.0101 (16)	0.0143 (16)	0.0078 (16)
N4	0.042 (2)	0.0298 (19)	0.034 (2)	−0.0031 (17)	0.0113 (17)	0.0018 (16)
N5	0.048 (2)	0.0330 (19)	0.0318 (19)	0.0080 (17)	0.0082 (17)	0.0027 (16)
C1	0.044 (3)	0.031 (2)	0.045 (3)	0.004 (2)	0.013 (2)	0.005 (2)
C2	0.045 (3)	0.043 (3)	0.036 (2)	−0.002 (2)	0.001 (2)	0.001 (2)
C3	0.044 (3)	0.040 (3)	0.035 (2)	0.006 (2)	0.001 (2)	0.004 (2)
C4	0.036 (2)	0.031 (2)	0.028 (2)	0.0082 (18)	0.0086 (18)	0.0045 (17)
C5	0.040 (3)	0.033 (2)	0.027 (2)	0.0048 (19)	0.0043 (19)	0.0013 (18)
C6	0.052 (3)	0.040 (3)	0.030 (2)	0.010 (2)	0.005 (2)	0.010 (2)
C7	0.046 (3)	0.038 (2)	0.033 (2)	0.013 (2)	0.014 (2)	0.011 (2)
C8	0.056 (3)	0.051 (3)	0.036 (2)	0.014 (2)	0.016 (2)	0.012 (2)
C9	0.060 (3)	0.048 (3)	0.038 (3)	0.014 (2)	0.018 (2)	0.018 (2)
C10	0.052 (3)	0.034 (2)	0.050 (3)	0.013 (2)	0.027 (2)	0.012 (2)
C11	0.044 (3)	0.030 (2)	0.036 (2)	0.0066 (19)	0.021 (2)	0.0063 (18)
C12	0.044 (3)	0.032 (2)	0.042 (3)	0.009 (2)	0.020 (2)	0.004 (2)
C13	0.039 (3)	0.027 (2)	0.038 (2)	0.0062 (18)	0.011 (2)	0.0052 (18)
C14	0.041 (3)	0.033 (2)	0.054 (3)	0.010 (2)	0.018 (2)	0.006 (2)
C15	0.040 (3)	0.032 (2)	0.060 (3)	0.007 (2)	0.003 (2)	0.004 (2)
C16	0.057 (3)	0.033 (2)	0.042 (3)	0.014 (2)	0.000 (2)	0.004 (2)
C17	0.036 (2)	0.032 (2)	0.032 (2)	0.0054 (18)	0.0080 (19)	0.0023 (18)
O3	0.059 (2)	0.053 (2)	0.052 (2)	−0.0049 (18)	0.0235 (18)	0.0038 (17)
N6	0.058 (3)	0.038 (2)	0.044 (2)	0.0050 (19)	0.021 (2)	0.0033 (18)
C18	0.063 (4)	0.070 (4)	0.063 (4)	0.018 (3)	0.022 (3)	−0.010 (3)
C19	0.104 (5)	0.049 (3)	0.080 (4)	0.024 (3)	0.048 (4)	0.023 (3)
C20	0.065 (3)	0.041 (3)	0.036 (3)	0.002 (3)	0.010 (3)	0.000 (2)

### Geometric parameters (Å, °)

**Table:**

Hg1—N5	2.315 (3)	C7—C8	1.386 (6)
Hg1—N1^i^	2.351 (3)	C8—C9	1.374 (6)
Hg1—Br1	2.5108 (8)	C8—H8	0.9300
Hg1—Br2	2.5289 (12)	C9—C10	1.383 (6)
O1—C6	1.218 (5)	C9—H9	0.9300
O2—C12	1.225 (5)	C10—C11	1.382 (6)
N1—C5	1.335 (5)	C10—H10	0.9300
N1—C1	1.337 (5)	C11—C12	1.503 (6)
N1—Hg1^i^	2.351 (3)	C13—C14	1.384 (6)
N2—C6	1.357 (5)	C13—C17	1.389 (5)
N2—C4	1.394 (5)	C14—C15	1.369 (6)
N2—H21	0.856 (10)	C14—H14	0.9300
N3—C7	1.334 (5)	C15—C16	1.370 (6)
N3—C11	1.342 (5)	C15—H15	0.9300
N4—C12	1.346 (5)	C16—H16	0.9300
N4—C13	1.414 (5)	C17—H17	0.9300
N4—H22	0.859 (10)	O3—C20	1.236 (6)
N5—C17	1.326 (5)	N6—C20	1.306 (6)
N5—C16	1.337 (6)	N6—C19	1.446 (6)
C1—C2	1.361 (6)	N6—C18	1.452 (6)
C1—H1	0.9300	C18—H18A	0.9600
C2—C3	1.374 (6)	C18—H18B	0.9600
C2—H2	0.9300	C18—H18C	0.9600
C3—C4	1.401 (5)	C19—H19A	0.9600
C3—H3	0.9300	C19—H19B	0.9600
C4—C5	1.390 (5)	C19—H19C	0.9600
C5—H5	0.9300	C20—H20A	0.9300
C6—C7	1.502 (6)		
			
N5—Hg1—N1^i^	103.47 (12)	C8—C9—H9	120.6
N5—Hg1—Br1	117.00 (9)	C10—C9—H9	120.6
N1^i^—Hg1—Br1	107.93 (9)	C11—C10—C9	118.8 (4)
N5—Hg1—Br2	102.81 (9)	C11—C10—H10	120.6
N1^i^—Hg1—Br2	100.41 (9)	C9—C10—H10	120.6
Br1—Hg1—Br2	122.54 (3)	N3—C11—C10	123.1 (4)
C5—N1—C1	118.4 (4)	N3—C11—C12	117.5 (3)
C5—N1—Hg1^i^	118.6 (3)	C10—C11—C12	119.4 (4)
C1—N1—Hg1^i^	122.0 (3)	O2—C12—N4	123.6 (4)
C6—N2—C4	126.9 (4)	O2—C12—C11	120.7 (4)
C6—N2—H21	116 (3)	N4—C12—C11	115.7 (4)
C4—N2—H21	118 (3)	C14—C13—C17	118.3 (4)
C7—N3—C11	117.1 (3)	C14—C13—N4	122.0 (4)
C12—N4—C13	122.6 (4)	C17—C13—N4	119.8 (4)
C12—N4—H22	116 (3)	C15—C14—C13	118.7 (4)
C13—N4—H22	121 (3)	C15—C14—H14	120.7
C17—N5—C16	118.9 (4)	C13—C14—H14	120.7
C17—N5—Hg1	118.2 (3)	C14—C15—C16	119.9 (4)
C16—N5—Hg1	122.4 (3)	C14—C15—H15	120.0
N1—C1—C2	121.6 (4)	C16—C15—H15	120.0
N1—C1—H1	119.2	N5—C16—C15	121.8 (4)
C2—C1—H1	119.2	N5—C16—H16	119.1
C1—C2—C3	121.0 (4)	C15—C16—H16	119.1
C1—C2—H2	119.5	N5—C17—C13	122.5 (4)
C3—C2—H2	119.5	N5—C17—H17	118.8
C2—C3—C4	118.2 (4)	C13—C17—H17	118.8
C2—C3—H3	120.9	C20—N6—C19	120.5 (4)
C4—C3—H3	120.9	C20—N6—C18	121.7 (4)
C5—C4—N2	117.6 (4)	C19—N6—C18	117.7 (4)
C5—C4—C3	117.2 (4)	N6—C18—H18A	109.5
N2—C4—C3	125.2 (4)	N6—C18—H18B	109.5
N1—C5—C4	123.5 (4)	H18A—C18—H18B	109.5
N1—C5—H5	118.2	N6—C18—H18C	109.5
C4—C5—H5	118.2	H18A—C18—H18C	109.5
O1—C6—N2	124.0 (4)	H18B—C18—H18C	109.5
O1—C6—C7	121.1 (4)	N6—C19—H19A	109.5
N2—C6—C7	114.9 (4)	N6—C19—H19B	109.5
N3—C7—C8	123.5 (4)	H19A—C19—H19B	109.5
N3—C7—C6	117.3 (3)	N6—C19—H19C	109.5
C8—C7—C6	119.2 (4)	H19A—C19—H19C	109.5
C9—C8—C7	118.7 (4)	H19B—C19—H19C	109.5
C9—C8—H8	120.7	O3—C20—N6	126.2 (5)
C7—C8—H8	120.7	O3—C20—H20A	116.9
C8—C9—C10	118.8 (4)	N6—C20—H20A	116.9
			
N1^i^—Hg1—N5—C17	−141.8 (3)	C6—C7—C8—C9	179.1 (4)
Br1—Hg1—N5—C17	99.7 (3)	C7—C8—C9—C10	−0.1 (7)
Br2—Hg1—N5—C17	−37.6 (3)	C8—C9—C10—C11	0.2 (7)
N1^i^—Hg1—N5—C16	46.8 (3)	C7—N3—C11—C10	−0.3 (6)
Br1—Hg1—N5—C16	−71.7 (3)	C7—N3—C11—C12	−179.8 (3)
Br2—Hg1—N5—C16	151.0 (3)	C9—C10—C11—N3	0.0 (6)
C5—N1—C1—C2	−1.1 (6)	C9—C10—C11—C12	179.5 (4)
Hg1^i^—N1—C1—C2	167.2 (3)	C13—N4—C12—O2	−4.8 (7)
N1—C1—C2—C3	1.6 (7)	C13—N4—C12—C11	174.0 (4)
C1—C2—C3—C4	−0.5 (7)	N3—C11—C12—O2	−169.3 (4)
C6—N2—C4—C5	−177.3 (4)	C10—C11—C12—O2	11.2 (6)
C6—N2—C4—C3	0.9 (7)	N3—C11—C12—N4	11.9 (6)
C2—C3—C4—C5	−1.1 (6)	C10—C11—C12—N4	−167.7 (4)
C2—C3—C4—N2	−179.4 (4)	C12—N4—C13—C14	51.7 (6)
C1—N1—C5—C4	−0.7 (6)	C12—N4—C13—C17	−128.8 (4)
Hg1^i^—N1—C5—C4	−169.4 (3)	C17—C13—C14—C15	−0.4 (6)
N2—C4—C5—N1	−179.9 (4)	N4—C13—C14—C15	179.2 (4)
C3—C4—C5—N1	1.8 (6)	C13—C14—C15—C16	0.5 (6)
C4—N2—C6—O1	−1.7 (8)	C17—N5—C16—C15	−1.0 (6)
C4—N2—C6—C7	179.3 (4)	Hg1—N5—C16—C15	170.4 (3)
C11—N3—C7—C8	0.4 (6)	C14—C15—C16—N5	0.2 (7)
C11—N3—C7—C6	−178.9 (4)	C16—N5—C17—C13	1.0 (6)
O1—C6—C7—N3	−177.7 (4)	Hg1—N5—C17—C13	−170.7 (3)
N2—C6—C7—N3	1.3 (6)	C14—C13—C17—N5	−0.3 (6)
O1—C6—C7—C8	3.0 (7)	N4—C13—C17—N5	−179.9 (4)
N2—C6—C7—C8	−178.0 (4)	C19—N6—C20—O3	2.1 (7)
N3—C7—C8—C9	−0.2 (7)	C18—N6—C20—O3	−179.2 (5)

Symmetry codes: (i) −*x*+1, −*y*+1, −*z*+1.

### Hydrogen-bond geometry (Å, °)

**Table:**

*D*—H···*A*	*D*—H	H···*A*	*D*···*A*	*D*—H···*A*
N4—H22···O3^i^	0.86 (1)	2.08 (2)	2.891 (5)	157 (4)
N2—H21···O3^i^	0.86 (1)	2.34 (2)	3.076 (5)	144 (3)
N2—H21···N3	0.86 (1)	2.25 (4)	2.685 (5)	111 (3)

Symmetry codes: (i) −*x*+1, −*y*+1, −*z*+1.
